# Eales disease in a young adult man Case report

**Published:** 2017

**Authors:** Dan Călugăru, Mihai Călugăru, Chabi El Ghali

**Affiliations:** * Department of Ophthalmology, University of Medicine Cluj-Napoca, Romania; ** OcuCenter Ophthalmological Clinic, Cluj-Napoca, Romania

**Keywords:** Eales disease, young adult, pars plana vitrectomy, endolaser, laser scatter retinal photocoagulation

## Abstract

****Purpose**:**

To report the case of a 39-year-old man with bilateral Eales disease.

****Methods**:**

The clinical, angiographic, and ultrasonographic evaluations of the patient were thoroughly and specifically carried out.

****Results**:**

The treatment consisted of pars plana vitrectomy and endolaser treatment at the time of surgery to the left eye and laser scatter retinal photocoagulation to the right eye. Postoperatively, the visual acuity improved at 20/ 20 to the left eye.

****Conclusions**:**

Eales disease is a clinical diagnosis of exclusion as many other retinal disorders can mimic it, especially conditions of retinal inflammation or neovascularization. The condition, with a characteristic clinical picture, fluorescein angiographic finding, and natural course, is considered a specific disease entity, easily distinguished from other vasculitides and retinopathies.

## Introduction

Eales disease is a primary idiopathic peripheral obstructive retinal vasculopathy of unclear etiology. The disease, also known as angiopathia retinae juvenilis, periphlebitis retinae, primary perivasculitis of the retina, is characterized by three overlapping stages of venous inflammation (peripheral retinal vessels first become inflamed and sheathed, then become occluded), ischemia, and retinal neovascularization, being hallmarked by recurrent retinal and vitreal hemorrhages [**1**]. The condition usually affects healthy young adult males in their second decade and is observed in men of all age groups being bilateral in up to 90% of the patients although symptoms often present unilaterally. Peak age of onset for Eales disease is 20-35 years, with a reported range of 13-63 years [**2**]. 

Herein, we present a case of bilateral Eales disease in a young adult man given the issues arising from the diagnosis and treatment of the disease. 

## Case presentation

**Fig. 1:**
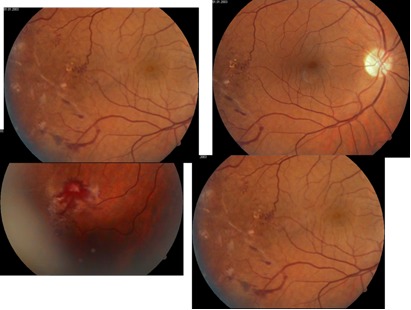
Ocular fundus of the right eye in a 39-year-old man with bilateral Eales disease. (Left and right top; right bottom) Pallor of the optic nerve head; temporally from the macula in the mid-retina periphery there are scatter wet perivascular exudates, dotted and flame-shaped intraretinal hemorrhages, focal occlusions of retinal vessels, extensive nonperfused areas of the capillaries giving rise to neovascularization; the affected vessels become dilated and tortuous and take on the form of a corkscrew; microaneurysms are observed in the patent capillaries located in the region of a vascular occlusion. (Left bottom) Preretinal fibrosis (epiretinal gliosis) located inferotemporally from the macula in the mid-retina periphery caused retinal tears due to contraction of the fibrovascular tissue

A 39-year-old man presented to the clinic with complaints of decreased and blurred vision, photopsia, and floaters in his left eye over the previous two weeks. The patient’s past medical history was unremarkable. The family history was noncontributory. His best-corrected visual acuity at the initial presentation was 20/ 20 in the right eye and hand movement in the left eye. Anterior segment and intraocular pressure were normal. The visual field using the Goldmann perimeter and the Humphrey static achromatic automatic perimetry (central 30-2 threshold test) was normally at the right eye and could not be performed to the left eye owing to the markedly decreased visual acuity. Ultrasonography of the left eye highlighted dense vitreous floaters, which obscured the view of the fundus. Dilated ocular fundus examination of the right eye (**Fig. 1**) showed pallor of the optic nerve head; temporally from the macula in the mid-periphery, there were perivascular sheathing, scattered wet perivascular exudates, dotted and flame-shaped intraretinal hemorrhages, focal occlusions of retinal vessels, extensive nonperfused areas of the capillaries from the macula in the mid-retina periphery, which extended towards the posterior pole and retinal periphery, giving rise to preretinal neovascularization prominent into the vitreous in the fashion of a sea fan.

**Fig. 2:**
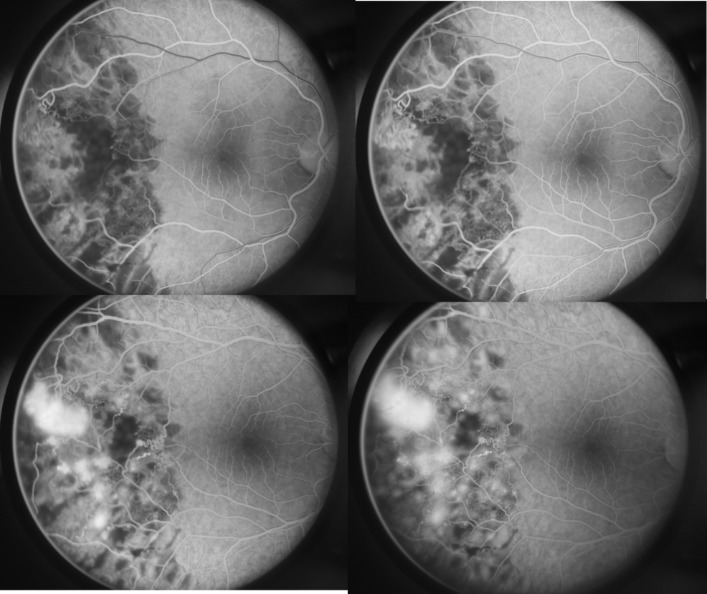
Fluorescein angiography of the right eye in a 39-year-old man with bilateral Eales disease (Left top) The arterial phase shows arterial filling and continuation of the choroidal filling, multiple hypofluorescent laser photocoagulation scars, minimal hyperfluorescence at the area of the newly formed vessels, and well-delineated areas of nonperfusion. (Right top) The arteriovenous (capillary) phase exhibits early laminar flow in veins, leakage of the sheated and newly formed vessels, telangiectasias, microaneurisms, sharply demarcated areas of nonperfusion, hypofluorescence of the photocoagulation scars persists but with slight staining of their margins coming from the adjacent choriocapillaries without leakage of the dye. (Left bottom) The venous phase displays bud-like dilatation of the remaining patent vessels, considerable leakage of the new-formed blood vessels in the form of a sea fan. (Right bottom) The late (elimination) phase demonstrates considerable leakage of the dye coming from the neovascular membrane, remarkable staining of the vessels, increased fluorescence of the photocoagulation foci

Neovascularization occurred at the junction between perfused and nonperfused retina. The affected vessels became dilated and tortuous and might take on the form of a corkscrew. Demarcation between perfused and nonperfused zones was marked by veno-venous shunts, venous beadings, and microaneurysms, which were observed in the patent capillaries located in the region of the vascular occlusion. Spontaneous chorioretinal scars occurred. Preretinal fibrosis (epiretinal gliosis) located inferotemporally from the macula in the mid-retina periphery caused retinal tears due to the contraction of the fibrovascular membrane. Fluorescein angiography (**Fig. 2**) showed leakage of sheathed vessels, retinal vascular tortuosity and telangiectasias, shunt vessels, venous stasis, capillary non-perfusion and sharply demarcated areas of non-perfusion; intensive staining in the late phases in the area of the newly formed vessels (budlike dilatation in the fashion of a sea fan). The pneumophtisiological examination (chest X-ray) including the Mantoux test was normally. Results of the serological testing were unremarkable.

**Fig. 3:**
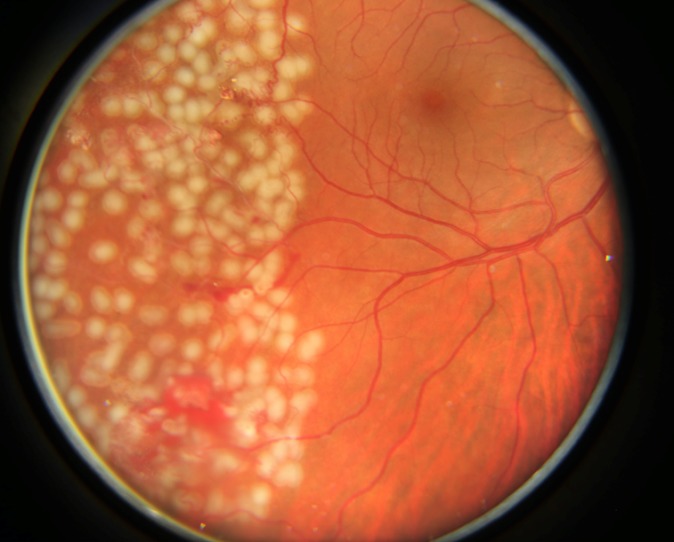
Foci of laser scatter retinal photocoagulation scars in the right eye of the 39-year-old man with bilateral Eales disease

**Fig. 4:**
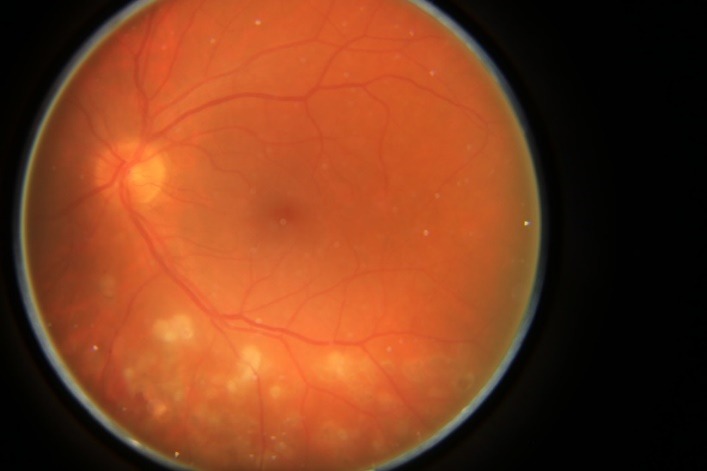
The left eye of the 39-year-old man with bilateral Eales disease exhibiting multiple photocoagulation scars after endolaser located inferiorly from the macula in the mid-retina periphery

Taking into account all the clinical, angiographic, and ultrasonographic assessments, the diagnoses of bilateral Eales disease in the late (proliferative) stage with neovascularization and retinal and vitreous hemorrhages were established. The treatment consisted of pars plana vitrectomy and endolaser treatment at the time of surgery to the left eye and laser scatter retinal photocoagulation to the right eye (**Fig. 3**). Left eye ocular fundus examination showed multiple chorioretinal scars after endolaser located inferiorly from the macula in the mid-retina periphery (**Fig. 4**). The visual acuity improved at 20/20 to this eye. 

## Discussion

As many other retinal disorders can mimic it, especially conditions of retinal inflammation or neovascularization, Eales disease is a clinical diagnosis of exclusion. With a characteristic clinical picture, fluorescein angiographic finding, and natural course, the condition is considered a specific disease entity, easily distinguished from other vasculitides and retinopathies (**Table 1**).

**Table 1:** Differential diagnosis of Eales disease

Vasculitides and retinopathies
Retinopathy of prematurity
Branch retinal vein occlusion
Branch retinal artery occlusion
Cytomegalovirus retinitis
Acute retinal necrosis
Bechet’s syndrome
Radiation retinopathy
Coagulopathy
Wegener’s granulomatosis
Lyme disease
Syphilis
Multiple sclerosis
Sarcoidosis
Birdshot retinochoroidopathy
Multifocal choroidopathy syndromes
Serpiginous choroiditis
Coat’s disease
Idiopathic retinal vasculitis
Aneurysms and neuroretinitis
Susac disease
Leukemia
Sickle cell retinopathy
Familial exudative vitreoretinopathy
Diabetic retinopathy
Retinal detachment
Exudative retinal detachment

The etiopathogenic diagnosis is one of presumption. The cause of this disease is not known. However, the Polymerase Chain Reaction detected the deoxyribonucleic acid of the bacterium Mycobacterium tuberculosis in a significant number of patients [**3**]. The pathophysiology of this disease is mostly unknown. Laboratory tests are neither sensitive nor specific for Eales disease, but may be helpful in detecting systemic causes of retinal vasculitis or peripheral retinal non-perfusion. Eales disease is believed to be a primary, noninflammatory disorder of the walls of peripheral retinal vessels, namely, the shunt vessels [**2**]. The association of Eales disease with both ocular inflammation and hypersensitivity to tuberculin protein suggests that this disease may be associated with immunologic phenomena whose mechanism remains unknown. Less commonly, it has been linked to central nervous system pathology (stroke, demyelination, and intranuclear ophthalmoplegia), hematological abnormalities (abnormal red blood cell morphology), vestibulo auditory dysfunction, and focal sepsis. The exact pathological mechanisms in Eales disease are largely unclear despite several basic science and clinical studies. Immunologic mechanisms, oxidative stress, and decreased vitamins C and E have been proposed. Similarly, an increased vascular endothelial growth factor results in retinal neovascularization [**4**].

Therapeutically, the treatment has to be adapted to the evolution stage of the disease. The steroidal and non-steroidal agents, intravitreal anti-vascular endothelial growth factor (VEGF) therapy [**3**-**5**], and anti-tubercular therapy (in patients with exposure to tuberculosis) are recommended in the early stages. For patients with a later stage of the disease, the laser scatter retinal photocoagulation (peripheral pan retinal photocoagulation) in the areas of retinal non-perfusion and direct photocoagulation of new and underlying feeder vessels were suggested. For nonresolving vitreous hemorrhages and/ or retinal detachment, the pars plana vitrectomy (no later than 6 months following the onset of hemorrhage) with or without other vitreoretinal surgical procedures is necessary.

## Conclusions

As many other retinal disorders can mimic it, especially conditions of retinal inflammation or neovascularization, Eales disease is a clinical diagnosis of exclusion. With a characteristic clinical picture, fluorescein angiographic finding, and natural course, the disease is considered a specific disease entity, easily distinguished from other vasculitides and retinopathies.

**Acknowledgments**

All the authors have made substantial contributions to the manuscript (i.e., data collection, analysis, writing, and editing assistance); they have provided the corresponding author with a written permission to be named in the manuscript. The authors do not have a financial relationship. No organization sponsored the research. 

**Disclosures**

The authors have no proprietary of commercial interest in any of the materials discussed in this article; they declare no conflict of interest. The authors have nothing to disclose.

## References

[1] Sen A, Paine SK, Chowdhury IH, Mukherjee A, Choudhury S, Mandal LK, Bhattacharya B (2011). Assessment of gelatinase and tumor necrosis factor-alpha level in the vitreous and serum of patients with Eales disease; role of inflammation-mediated angiogenesis in the pathogenesis of Eales disease. Retina.

[2] Das T, Pathengay A, Hussain N, Biswas J (2010). Eales disease: diagnosis and management. Eye.

[3] Madhavan HN, Therese KL, Doraiswamy K (2002). Further investigations on the association of Mycobacterium tuberculosis with Eales disease. Indian J Ophthalmol.

[4] Patwardhan SD, Azad E, Shah BM, Sharma Y (2011). Role of intravitreal bevacizumab in Eales disease with dense vitreous haemorrhage: a prospective randomized control study. Retina.

[5] Călugăru D, Călugăru M (2016). Combination of peripheral laser photocoagulation with intravitreal bevacizumab in naïve eyes with macular edema secondary to CRVO: prospective randomized study. Eye.

